# Historical Evolution and Food Control Achievements of Near Infrared Spectroscopy, Electronic Nose, and Electronic Tongue—Critical Overview

**DOI:** 10.3390/s20195479

**Published:** 2020-09-24

**Authors:** Balkis Aouadi, John-Lewis Zinia Zaukuu, Flora Vitális, Zsanett Bodor, Orsolya Fehér, Zoltan Gillay, George Bazar, Zoltan Kovacs

**Affiliations:** 1 Department of Measurement and Process Control, Faculty of Food Science, Szent István University, H-1118 Budapest, Hungary; aouadi.balkis@hallgato.uni-szie.hu (B.A.); zaukuu.john-lewis.zinia@hallgato.uni-szie.hu (J.-L.Z.Z.); vitalis.flora@hallgato.uni-szie.hu (F.V.); bodor.zsanett@hallgato.uni-szie.hu (Z.B.); gillay.zoltan@szie.hu (Z.G.); 2 Institute of Agribusiness, Faculty of Economics and Social Sciences, Szent István University, H-2100 Gödöllő, Hungary; feher.orsolya@szie.hu; 3 Department of Nutritional Science and Production Technology, Faculty of Agricultural and Environmental Sciences, Szent István University, H-7400 Kaposvár, Hungary; bazar@agrilab.hu; 4 ADEXGO Kft., H-8230 Balatonfüred, Hungary

**Keywords:** sensors, food authenticity, food adulteration, chemometrics, fingerprinting

## Abstract

Amid today’s stringent regulations and rising consumer awareness, failing to meet quality standards often results in health and financial compromises. In the lookout for solutions, the food industry has seen a surge in high-performing systems all along the production chain. By virtue of their wide-range designs, speed, and real-time data processing, the electronic tongue (E-tongue), electronic nose (E-nose), and near infrared (NIR) spectroscopy have been at the forefront of quality control technologies. The instruments have been used to fingerprint food properties and to control food production from farm-to-fork. Coupled with advanced chemometric tools, these high-throughput yet cost-effective tools have shifted the focus away from lengthy and laborious conventional methods. This special issue paper focuses on the historical overview of the instruments and their role in food quality measurements based on defined food matrices from the Codex General Standards. The instruments have been used to detect, classify, and predict adulteration of dairy products, sweeteners, beverages, fruits and vegetables, meat, and fish products. Multiple physico-chemical and sensory parameters of these foods have also been predicted with the instruments in combination with chemometrics. Their inherent potential for speedy, affordable, and reliable measurements makes them a perfect choice for food control. The high sensitivity of the instruments can sometimes be generally challenging due to the influence of environmental conditions, but mathematical correction techniques exist to combat these challenges.

## 1. Introduction

According to the World Food Summit, “Food security exists when all people, at all times, have physical and economic access to sufficient, safe and nutritious food that meets their dietary needs and food preferences for an active and healthy life” [1]. Naturally, food is a global necessity generally consumed for pleasure, energy, health, or wellbeing but can, ironically, also infringe upon all these benefits or even present dire consequences when not in the appropriate quality. The 21st century has, however, been plagued by many food quality concerns ranging from malnutrition, food hazards, to food fraud. To mitigate the consequences of these threats, many interventions have been introduced by diverse regulatory authorities such as the Food and Agricultural Organization (FAO), Codex Alimentarius, and the Foods and Drugs and Agency in numerous countries. Nonetheless, food hazards and food fraud continue to exist in the food industry due to many reasons, including urbanization and the complexity of the food chain system. Food hazards refer to the intentional or unintentional existence of anything in food that can cause harm upon consumption and it cuts across physical, chemical, or microbiological hazards. Food fraud, on the other hand, is the intentional or deliberate attempt to trick consumers by mislabeling or with inferior alternatives of the food product. It is considered to be the second most significant black-market activity in the European Union after drug trafficking. However, the unprecedented rates of changing trends and heightened fraud claims sparked demand for swifter analytical tools. Many types of fraud have been reported for many foods ranging from powdered foods [2,3] to both nonalcoholic and alcoholic drinks [4]. Under its varying shapes and forms, the effects of fraudulent practices have been shown to expand beyond threatening human and animal health to undermining the global economy. For instance, an estimated $55 billion worth of food is counterfeited worldwide every year. In the United States alone, the damage caused by food counterfeiting equals 7% of the Gross Domestic Product (GDP) [5]. Adding to the economic and health burden of food fraud, losing the consumer’s trust is another major concern in the food industry.

These detrimental effects on the consumer’s trust have been equally corroborated by [6], who proved that both conscious consumers and those who have already encountered food counterfeiting expressed lower trust levels in the authorities or food companies. While the respondents’ gender and income level did not have any significant effect on how risks are perceived, the level of education has been a major determining factor. Thus, the higher one’s education, the more likely they were to consider imported foods to be risky due to food counterfeiting. These studies have also shown that informed and quality conscious consumers can assess more of those products coming from a reliable source and can even detect if the property of a given product differs from normally expected parameters. What these observations confirm is that having access to safe, credible, and traceable information regarding food production and distribution is in the common interest of all actors in the food economy.

In the fight against acts of counterfeiting, numerous domestic and international initiatives have been launched. Among many others, Operation OPSON, coordinated by EUROPOL and INTERPOL, involves 61 countries and focuses on counterfeit food and drink. As per the targeted products, they range from commonly consumed products such as olive oil, various beverages, and mineral waters to luxury goods such as caviar [7].

Besides these initiatives, mitigating food fraud and ensuring food quality called for the joint effort of food practitioners and academia. The latter adopted two approaches, one consists of deploying traditional analytical techniques, the other implements modern alternatives. In modern analytical techniques, a new trend is to use nondirect properties of the analyzed sample and use these properties in a correlative way to predict various quality attributes of the tested materials. Commonly used analytical methods successfully employing this approach in food quality determination are near-infrared (NIR) spectroscopy, electronic nose (E-nose), and electronic-tongue (E-tongue). In the following sections, a historical overview of these three rapid correlative analytical methods and their comparison to conventional ones will be elucidated. Furthermore, the major potentials and challenges encountered when applying these techniques in the food industry will be summarized.

## 2. Correlative vs. Conventional Analytical Methods in Food Quality Assessment

In the generally accepted food quality evaluation protocols and early responses to food counterfeiting, major assessments of fraudulently manipulated products have been largely based on standard methods. In fact, amid rising concerns on whether the labels reflect the true state of consumed products or not, these detection methods have been as diverse as the studied adulterants and their targeted food matrices. Existing literature categorizes these methods into chromatographic, isotopic, enzymatic, spectroscopic, elemental, sensorial, and proteogenomic analysis, etc. [8,9].

Given their marked ability to detect specific components of interest and to provide reliable results, these laboratory methods remain, to date, routinely used for qualitative purposes.

However, amidst the rapidly growing complexity of the food chain, the daunting challenges of guaranteeing food safety are also being magnified by the over-reliance on conventional techniques. For the most part, some of these conventional techniques hinder the advancement necessary for keeping up with the sophisticated ways of adulterating food. In the case of conventional analytical methods, a priori knowledge is essential, since the target chemical or physical property has to be known to detect and measure it. Furthermore, conventional methods are dedicated to single properties, whereas, correlative techniques may be applied globally in a holistic approach, without knowing which parameter would change during a process but, allow the determination of several compounds and properties at once. Assuredly, up to recent days, the determinations of both the quality and potential adulteration of various food products have become so intricate that traditional tools are no longer able to keep track of them. With these underlying limitations, automating analysis is poised to play a crucial role in addressing the untapped aspects of food fraud tracking systems and in food quality characterization. By incorporating the correlative analytical techniques along with sophisticated mathematical evaluation methods, major impairments afflicting the results obtained by conventional techniques can no longer be overlooked and big data loads can be handled. Other requirements of highly qualified analysts and matrix specific reagents can also be dispensable [10]. Table 1 shows some of the main characteristic attributes of correlative analytical techniques, namely NIR spectroscopy, E-noses, and E-tongues in comparison to some of the conventional methods commonly used in food authentication.

As presented in Table 1, these rapid and correlative analytical methods offer a set of technical advantages that permit the analysis of more complex matrices with enhanced accuracies, at swifter response rates [10,15]. Owed to their minimal preparation requirements and advanced designs, their integration into routine applications can be translated into higher performances. Nevertheless, to extend their application to wider scopes, a prerequisite knowledge of their limitations is also recommended. In this regard, even minute compositional differences of the studied products can cause interferences within the responses of the array of E-tongue and E-nose sensors [16]. Other reported disadvantages include the fluctuation of the sensor’s sensitivity due to external factors such as temperature and other environmental conditions, or surface defects emanating from cross-contamination. The sensor drift, engendered by such factors, has been a subject of a wide number of studies. With varying outcomes, the proposed solutions ranged from maintaining the cleanliness of the sensor membranes [17] to performing systematic calibrations and other mathematical corrections [18]. Analysis with NIR spectroscopy, on the other hand, can be dependent on reference methods, for calibration and validation purposes [19]. From an analytical standpoint, the quality of the obtained results is dictated by the variability induced by environmental conditions and the efficiency of compensating their resulting errors. The complexity of NIR spectra is another inevitable challenge [20] that demands a mastering of chemometric tools to understand any correlative relation between the generated spectra and the peculiarities of the studied samples.

**Figure 1:**
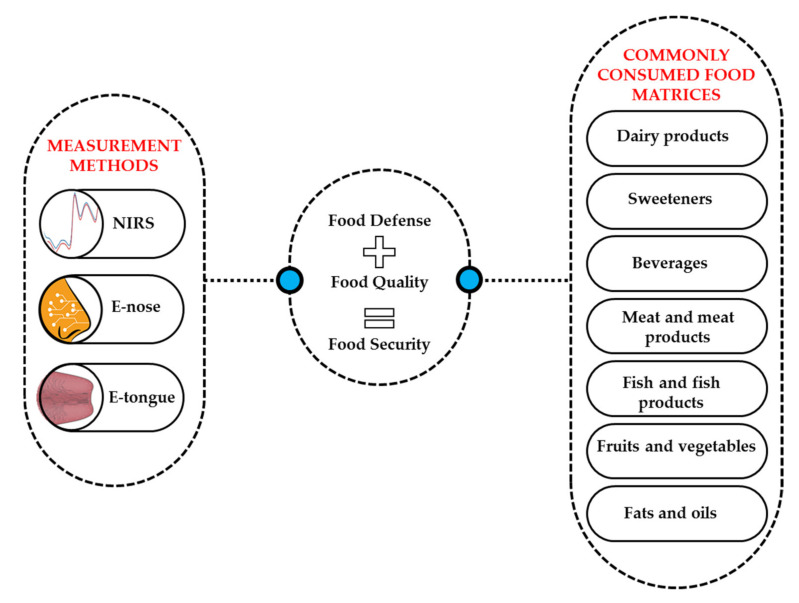
Correlative analytical methods used as food quality control devices, their applications, and some of the commonly consumed food matrices defined by the Codex General Standard for Food Additives [21].

Throughout the following sections, prominent data analysis tools and applications of NIR spectroscopy, E-nose, and E-tongue in the domain of food quality assessment are discussed based on commonly consumed food matrices defined by the Codex General Standard for Food Additives [21] (Figure 1).

### Data Analysis

Extracting valuable information related to chemical compositions from the data generated by correlative analytical methods, such as NIR spectroscopy, E-nose, and E-tongue, demands a set of special mathematical tools called chemometrics. In a definition by [22], “Chemometrics utilizes mathematical and statistical modelling to recognize patterns and relationships within highly complex data and translates them into useable analytical parameters.” Therefore, chemometrics is inherently an interdisciplinary science, using mainly mathematical methods such as multivariate statistics (e.g., pattern recognition or fingerprinting), in combination with a priori knowledge of chemical, physical, or organoleptic properties. These tools are used for explorative analysis, classification, and predicting qualitative information [23]. The use of the mathematical statistics is often never devoid of the reference knowledge to understand the real behavior of correlative techniques for specific features of food products [24]. The mathematical tools range from classical to emerging, from linear to nonlinear, from transparent to “black box” type, from supervised to nonsupervised.

Essentially, what differentiates supervised techniques from nonsupervised ones is the reliance on predetermined dependent variables for the prediction of independent variables. The yielded model is subsequently used to categorize newer unlabeled samples. Unsupervised exploratory methods, on the other hand, recognize patterns among the different features or samples and create clusters without demanding dependent variables for modelling purposes [25,26].

Since the appearance of the correlative analytical methods, they have been capable of producing highly multidimensional and multicollinear data in conjunction with chemometrics [27]. This was mainly catalyzed by the immense progression of computer technology. In this regard, the principal component analysis (PCA) was the most basic qualitative explorative analysis. However, others such as discriminant analysis (DA), soft independent modelling of class analogies (SIMCA), one class classifier (OCC), partial least squares-discriminant analysis (PLS-DA), and k-nearest neighbors algorithm (KNN) have also evolved. Multiple linear regression (MLR), stepwise MLR, principal component regression (PCR), and partial least squares (PLS) regression have also been used for quantitative purposes. More sophisticated pattern recognition algorithms such as support vector machine (SVM), and the biologically inspired artificial neural network (ANN) and fuzzy logic are currently on the rise [28,29,30,31].

For many years, the professional explanations of a calibration on compositional variables arrived with chemical interpretations, with detailed chemometrics analysis. Nowadays it is more and more common that reliable validations of a model of any kind means more for the users than interpretive modelling. In this sense, the “black box”-type nonchemometric multivariate data analytical techniques could be explored but with caution. Artificial neural network (ANN) is one of the most emerging and promising techniques. The parameters of classification and prediction cannot be interpreted with the sensor signals, making it difficult to understand the underlying relationships [32]. Thus, ANN is a typical example of a “black-box” solution, and it might be useful when the “why?” is not important, but only the output is. It is preferably used when the database represents complex and nonlinear relationship between the measured parameters and the features to be predicted and its effectiveness is undeniable. ANN has also been acknowledged for the ability to deliver an output even when incomplete information was used during training. However, the reliability of its performance heavily depends on how important the missing values are [33] and because of its nonlinearity the prediction can be extraneously false for samples with a somehow unique measurement response.

Besides these statistical techniques, the data provided by any of the discussed correlative analytical methods require special pretreatments based on their unique behavior, data structure, and issues of sensitivity that can result from numerous environmental conditions. In this regard, noise reduction algorithms [34], drift corrections [18], normalizations, sensor/spectra selection, and data dimension reduction can be applied before using it to build multivariate models for qualitative and quantitative evaluations [35].

NIR spectroscopy, described in the next section, has the longest history with chemometrics [36] and has very simple principles and solutions which can be transferred to deal with challenges of electronic nose and tongue. Some books [37] and articles [31] have described these methods in deep detail [38] or from the practical perspective [39]. However, there is still a gap between mathematical-statistical sciences and the other, more experiential sciences, so a more interdisciplinary approach and collaboration may be necessary for the future [24].

## 3. Near Infrared (NIR) Spectroscopy: Historical Background and Food Quality Assessment

Herschel’s discovery of the near infrared energy back in 1800 marked the beginning of a novel era of experimentation, without which, elucidating basic phenomena would not be possible [40]. This conceptual knowledge later translated into industrial applications that were first initiated by Karl Norris in agriculture-related applications. In his pioneering study, near infrared (NIR) spectroscopy enabled the analysis of agricultural commodities, namely by determining the protein content of wheat [41].

Covering the range 780–2500 nm of the electromagnetic (EM) spectrum, NIR spectroscopy captures samples’ spectra in reflection, interactance, and transmission modes, thus providing rich information regarding the vibration of their elemental molecular bonds [42]. Upon interacting with the EM radiation, the captured spectra comprise both overtones and combination bands involving mainly hydrogen-bonded functional groups (–CH, –NH, –OH –SH). Such broad bands are highly informative and demand chemometric tools to disclose the correlation between spectral information and the structural features of the samples [43].

**Figure 2:**
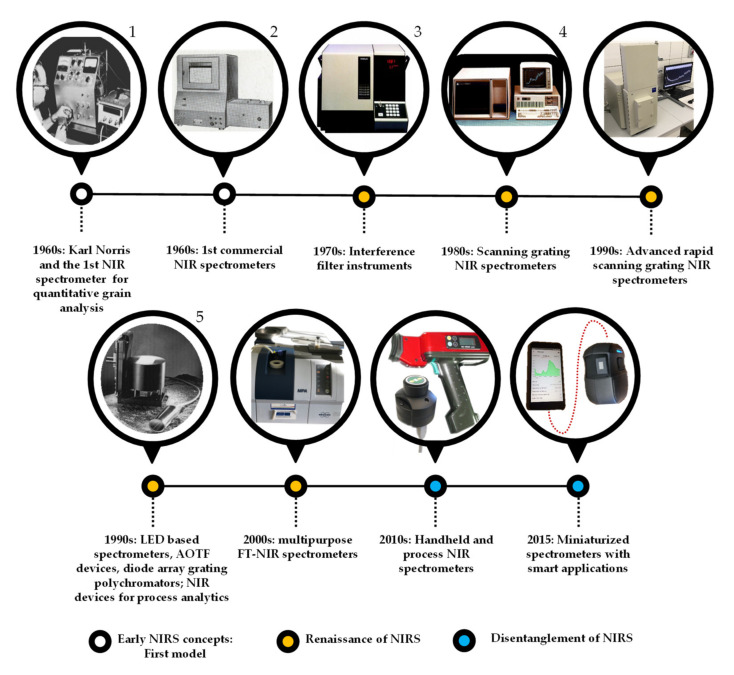
Overview of the evolution of near infrared spectrometers. Reproduced with permission from K H Norris [41] published by Journal of Near Infrared Spectroscopy (2020)^1^, K H Norris (2008)^2,3^ Technicon Instruments Corporation [44] published by Analytical Chemistry (2020)^4^, and J Malinen [45] published by Sensors and Actuators B (2020)^5^. The rest of the figures are self-developed based on authors’ own pictures.

A possible historical overview of the evolution of near infrared spectrometers with the main milestones is illustrated in Figure 2.

The first scanning spectrometers that employed NIR light were equipped with prism-grating monochromators, covering the UV-VIS-NIR range from 185 to 870 nm. Though, for many years during the renaissance of NIR spectroscopy, interference filter instruments were the most used. Still, in the late 1990s, 85% of instruments used for routine monitoring were estimated to be filter instruments [36]. The great advancements of the 1990s in electronics, including detectors and computer technology, resulted in the rise of the rapid scanning grating monochromators, which became the ultimate choice for both research and applications.

As a result of the upsurge of Raman spectroscopy, the development of FT-NIR (Fourier transform NIR) instrumentation came about in the 1990s [36]. First, traditional mid-IR spectroscopic companies provided conversions of their classical mid-IR instruments, but later launched the production of NIR dedicated FT instruments. These developments met a silent but significant market need coming from analytical chemists who were more familiar with FT-IR instrumentation used in the analytical IR, thus, were more apt to open to the new field of NIR spectroscopy—combined with often demonized multivariate data analysis—when at least the instrumentation was known. In the early 2000s, the agri-food sector was dominated by benchtop grating spectrometers, while FT-NIR spectrometers started to spread in pharmaceutical applications, where their narrow bandpass meant an advantage, even with the compromise of a lower signal-to-noise ratio. After several market changes and instrument developments, the above-mentioned barriers are rarely present, and both types of devices can be found in both fields, agri-food or pharma.

Regarding the detectors, silicon (Si) is still widely used for short-wave NIR (780–1100 nm), and lead sulphide (PbS) has been changed to indium gallium arsenide (InGaAs) that is less sensitive to temperature when used in the 800–1700 nm range, allowing cost-effective applications. The addition of optical fibers to the spectrometers allows collecting spectral information among conditions that are ineligible for highly precise and sensitive instruments.

The versatility in the design of NIR spectrometers has been further extended through gradual improvements aimed at enhancing the functionality of NIR spectroscopy resulting in a plethora of instruments [20]. The most recent and most significant achievement of NIR spectroscopy was the miniaturization, which undoubtedly resulted in the disentanglement of the technology [46]. The palm-sized or even smaller devices, containing e.g., linear variable filter with photodiode detector array, acousto-optic tunable filter, or micro-electro-mechanical systems with single photodiode detector, combined with advanced data transfer technologies, have opened new fields for applications, both in terms of handheld end-user devices and process analytical tools [47]. It became quite normal to be able to purchase a NIR scanner for personal use, and it is common to include a NIR device in an industrial process where quality has to be monitored or optimized. We are now in the era of ex-laboratory applications of NIR spectroscopy.

Multivariate data analysis of NIR spectroscopy has also undergone huge developments [48], sometimes in combination with the hardware. Karl Norris used scanning devices at USDA that allowed the spectroscopic inspection of the spectral data, instead of the purely statistical approaches that could be used by those having access to filter devices measuring at some discrete wavelengths. Having this advantage, Norris developed his derivative calculation method for data treatment in combination with a parsimonious interactive multiple regression based on sums and ratios of different wavelength channels. This was, particularly, to decrease additive and multiplicative effects caused by light scattering and path length variations [49]. Since derivative was not applicable for discrete spectral values of an instrument having 19 fixed wavelength filters, Harald Martens had to develop another correction method to overcome the burden of light scattering [50]. This is why nowadays we can use multiplicative scatter correction (MSC) in our calculations [51]. The regression methods started from multiple linear regression, when some discrete values were applied, and later, when scanning spectrometers generated a large amount of data, data reduction techniques became necessary. Principal component analysis turned to be the ultimate method to describe multidimensional variation and patterns, and to reduce the number of variables [52]. The most used quantitative linear calibration method is the partial least squares (PLS) regression [53]. With the decreased limitations in scanning speed, it became easy to collect thousands of spectra and create a large training database, which allows using other methods such as artificial neural network (ANN) or support vector machine (SVM). The SVM may be a better option when working with high dimensions but is very sensitive to outliers and noisy data and sometimes underperforms when large datasets are involved [31]. ANN has also been acknowledged for the ability to deliver an output even when incomplete information was used during training.

Just as on the side of spectrometers, users now have a vast variety of software and multivariate data analytical tools, starting from spectral pretreatments, through simplified data analysis, to detailed chemometrics when spectral information is translated to real analytical information. Push-button applications where an end-user can get feedback easily and rapidly are also continuing to evolve. With the progress in spectroscopic and IT hardware, tiny devices can do the scanning and hidden computers can deliver the results in any form within a blink of an eye. The only question remains: is that result good enough? Care must be exercised because in many cases little spectroscopic and sample handling knowledge is still necessary to avoid false assumptions. Nonetheless, the analytical advantages of NIR spectroscopy have been widely explored in the food industry.

### 3.1. Dairy Products

In inspecting the quality of dairy products, NIR spectroscopy was used in a wide spectrum of analysis. Research efforts were centered around monitoring the production of feta and Grana Padano cheeses and showed that using a discrete filter-based NIR analyzer, NIR could give a precise and direct representation of both production processes [54,55]. In another study, a scanning monochromator (SMC) with a fiber-optic probe was used to classify cheeses from different milk mixtures of cow, ewe, and goat milk, and their ripening time. When using the scores of the PCA for the artificial neural network model, a 100% accuracy was obtained for the classification of the ripening of the cheese samples. The classification according to the milk mixtures, however, was not as efficient with only 50% accuracy [56].

NIR light backscatter using a fiber optic sensor was also applied to monitor the incubation of the yoghurt. The results of the study highlighted the correlation between the changes of the spectra and pH of the samples (R^2^ > 0.99), indicating the adequacy of NIR as an alternative tool to pH meters during yoghurt incubation [57].

Application of NIR spectroscopy for detecting adulteration of different dairy products is also an immensely studied field. A study by Kamboj et al. [58] deployed an SMC spectrometer in the wavelength range of 700–2500 in reflectance mode to quantify added water in milk. Their results showed R^2^ values higher than 0.9 for the prediction of adulteration level.

Another reported application consisted of analyzing grated cheese adulterated with microcellulose, silicon-dioxide, wheat flour, wheat semolina, and sawdust using FT-IR with NIR module. The results showed that NIR was able to discriminate adulterated samples from nonadulterated ones with a classification accuracy of the PLS-DA models that is higher than 90% [59].

### 3.2. Sweeteners Including Honey

Authenticating organic sugars from those originating from other conventional sources is another major field of application of NIR spectroscopy.

Bázár et al. applied a benchtop NIR spectrometer with SMC, coupled to aquaphotomics for the classification and prediction of sugars in low concentration glucose, fructose, and lactose solutions obtaining R^2^ in the range of 0.7–0.99 depending on the wavelength range and sugar type [60].

Tahir et al. used the fusion of NIR and Raman-spectroscopy for the prediction of p-hydroxybenzoic acid (PHBA) content of honey and found strong correlation between the fused data and the PHBS content [61].

A handheld spectrometer with linear variable filter was used in diffuse reflectance mode in the wavelength range of 900–1650 nm, for the discrimination of crystal, demerara, and brown sugars with PLS-DA resulting in R^2^ < 0.99 [62].

Hungarian researchers analyzed linden, acacia, chestnut, and multifloral honeys and their mixtures using a benchtop NIR spectrometer and found average recognition and prediction abilities of 95.95% and 92.61%, respectively, using LDA for the botanical origin classification. For the geographical origin identification, 99.13% and 95.65% LDA accuracies were found [63].

Italian researchers also analyzed honey samples and built models using NIR-Flex 500 spectrometer and Raman spectroscopy for the botanical origin identification and revealed an accuracy amounting to 79% in the case of the NIR measurement [64].

An FT-NIR spectrophotometer was applied for the detection of adulteration in honey using glucose/fructose solutions and revealed the efficiency of NIR in the differentiation of authentic and adulterated samples with 95% recognition accuracy [65].

Bázár et al. [66] analyzed honey adulteration with HFCS syrups using benchtop SMC spectrometer and the aquaphotomics aspect of NIR and revealed the changes in the honey water structure as an effect of syrup addition. In this regard, the addition of syrup resulted in higher absorbance in the range of less H-bonded water (1320–1420 nm).

Similar results were obtained in another study using rice syrup as an adulterant of linden honey using benchtop double beam SMC instrument [67].

In a study of honey adulteration, authentic samples counterfeited with rice, beet, and corn, maltose, and high-fructose corn syrup and sucrose syrup in the range of 10–60% were analyzed with a SMC spectrometer and rapid content analyzer module for reflectance measurements, and with an ATR-FTIR spectrometer. The fusion of the two methods provided better accuracies (>90%) than those obtained using the two methods separately [68].

Benchtop SMC NIR spectrometer was also proven efficient when other adulterants comprising rice, brown cane, invert sugar, and fructose syrup in a concentration range of 5–40% were artificially added to honey and subjected to spectroscopic analysis. This study revealed correct classification of the samples with R^2^ values higher than 0.98 for the prediction of the syrup concentration [69].

Italian researchers applied a low level of heat treatment (39 °C for 30 min) and overheating (55 °C for 24 h) on three types of honey. After recording their NIR spectra using a benchtop SMC instrument, the results showed distinctive characteristics of the three types of honey in terms of their respective spectral pattern, however, the low level heat treatment did not affect the spectra of the samples [70].

Similar results were obtained in other studies using handheld instruments in the spectral range of 900–1700, where heat treatment of the samples (especially above 50 °C) affected the spectra of the samples and contributed to the determination of this rather widely spread kind of fraud [71,72].

### 3.3. Beverages

Beverages cover a very wide range of drinks providing a huge potential for the applications of NIR spectroscopy in the characterization of their quality from the most various aspects.

- Coffee

From determining coffee origin to evaluating the quality attributes, resorting to NIR spectroscopy has been extensively reported in the literature. Marquetti et al. [73], for instance, deployed a benchtop NIR spectrometer and recorded the spectra of coffee samples to assess their genotypic and geographical origin. After multiplicative scatter correction and 2nd derivative, the results of the PLS-DA enabled the correct discrimination of the geographical origin of the samples with 94.4% accuracy. In another study, diffuse reflectance spectra of different green coffee samples were analyzed using a benchtop NIR instrument and helped to predict the sensory properties (acidity, bitterness, flavor, cleanliness, body, and overall quality) of the samples with R^2^ coefficients exceeding 0.84 [74]. Other studies were aimed at screening the characteristics of coffee such as measuring the color and Arabica content using FT-NIR spectroscopy with R^2^ > 0.9 [75,76] as well as classifying Robusta and Arabica coffee samples according to the quality of the cup with the help of ATR-FTIR instrument. What these results have shown is that NIR spectroscopy could be used efficiently for the quality control of coffee.

- Tea

Instant green teas were analyzed using benchtop and portable NIR devices, the results showed that these instruments could be used to determine the main quality components of tea. The results comparing the benchtop and portable device showed, in the case of the prediction of total catechin, that the portable device provided higher R^2^ values and lower prediction error, while in the case of the caffeine better results were obtained using the benchtop instrument [77]. Matcha tea, another trending product, was also analyzed with portable NIR instruments and satisfactory results were obtained for the estimation of the polyphenol and amino acid content of the tea samples after choosing the most accurate pretreatment methods. The best results consisted of R^2^ of 0.86 for the prediction of polyphenols and 0.96 for the determination of amino acids [78].

- Fruit juices

Citric and tartaric acids were assessed and predicted with good precision (R^2^ > 0.98) and low errors (RMSECV < 0.62) when a handheld SMC spectrometer in reflectance mode was used [79]. This substantiates the importance of NIR spectroscopy for rapid monitoring of additives and preservatives in fruit juices when either transmittance, reflectance, or diffuse-reflectance mode is used. In combination with standard analytical techniques, both a benchtop and handheld SMC NIR in reflectance mode could discriminate different varieties of green and orange fleshed melons with 100% classification accuracy in LDA and predict multiple sensory parameters in PLS regression with high precision (R^2^ up to 0.96) and low errors (RMSECV as low as 0.24 g/mL) after cross-validation [80]. Transmittance mode benchtop FT-NIR provided high R^2^ in cross-validation (up to 0.94) and low errors (RMSECV as low as 5.07 mg nitrogen/L) when it was used to measure total yeast assimilable nitrogen, free amino nitrogen, and ammonia in 900 grape juice samples from 28 cultivars over three seasons [81]. This suggests that NIR can provide juice makers with the opportunity to make timelier and more informed nutrient supplementation decisions to achieve the desired juice style or quality.

- Soft drinks

Glucose, fructose, and sucrose contents of commercial soft drinks could also be rapidly predicted with precision (R^2^CV) higher than 0.91 and errors lower than 1.0 g/mL [82] when reflectance spectra from a benchtop SMC spectrometer were analyzed. This suggests a rapid approach for sugar content determination in soft drinks.

Reflectance NIR spectra from a handheld SMC instrument were used to fingerprint soluble solids contents of tea soft drink with a cross-validation R^2^ as high as 0.98 [83].

Furthermore, NIR proved to be a viable option in monitoring caffeine levels in soft drinks when more than 100 soft drinks were accurately classified and predicted with a benchtop FT-NIR spectrometer in transmission mode [84].

Ascorbic acid content of powdered soft drinks was determined and predicted with high accuracies after reflectance measurement with a benchtop FT-NIR [85]. This is particularly important because powdered soft drinks are often fortified with antioxidants such as ascorbic acid and, therefore, normally controlled by titration or chromatographic methods which can be both expensive and time-consuming.

- Mineral water

Three types of commercial mineral water, ultra-pure water, and deionized water were successfully discriminated with a benchtop SMC spectrometer in transflectance mode. It was revealed that the original spectra of water were more suitable than the second derivative spectra in discriminating the different waters with more 95% variance in PCA [86]. This emphasizes the importance of NIR spectra pretreatment techniques in optimizing results.

Aquaphotomics operates on the principle of using water as a holistic marker to extract information about many different water molecular conformations and their interaction with surrounding solutes through their absorbance bands and a light–water phenomenon [87]. Using NIR spectroscopy in combination with the aquaphotomics approach, seven commercial mineral waters and tap water were discriminated based on spectra variations using a mini SMC spectrometer in transmission mode [88].

This approach has been used in other studies regarding water such as the monitoring of water quality where aqueous solutions of acetic acid, lactose, and sodium chloride could be characterized by specific wavebands [89], using a benchtop SMC spectrometer.

### 3.4. Meat

NIR spectroscopy has unlimited potential for meat quality assessment because of its noninvasive analytical advantages.

Among many others it has been shown that NIR spectroscopy can rapidly detect freshness associated compounds in oxen [90], total viable count in pork [91,92], and bos grunniens meat [93].

Using a benchtop SMC spectrometer protein and intramolecular fat content of rabbit hind meat was determined with an R^2^ of 0.89 and 0.85, respectively, for fresh samples. Freeze-dried samples showed better accuracies with R^2^ of 0.99 (for fat) and 0.95 (for protein) [94].

A benchtop SMC spectrometer was used to predict meat chemical and fatty acid (FA) composition from 63 steers fed with sunflower or flaxseed in combination with high forage diets. Crude protein, moisture, fat content, saturated, monounsaturated, and branched FA and conjugated linoleic acid content were predicted with high precision (R^2^ as high as 0.97) and accuracy (RMSEC lower than 0.98 mg/g) [95]. These findings show the suitability of NIR spectroscopy for screening meat and meat-based products based on the content of beneficial FAs to human health.

Good calibrations were built for the total lipid content of beef resulting an R^2^ of 0.95 and SECV of 0.25 in cross-validation, when a SMC benchtop spectrometer was used [96].

For quality parameters determination purposes, NIR spectroscopy could predict sodium content in commercially processed meat [97] and in vacuum dried ham slices [98]. Other parameters such as ash and dry matter in freeze-dried ostrich meat were also studied [99].

Drip loss, color, and pH of 131 commercial pork loins were also predicted with high accuracies when NIR spectroscopy was used by Kapper et al. [100]. Similar studies also reported a good classification accuracy for pork tenderness (72%) and juiciness (73%) when a spectrometer with a bifurcated optical cable that resulted in a single reading from 400 to 1395 nm was used [101].

For food authentication, a benchtop SMC NIR spectrometer could detect the adulteration of minced beef with turkey meat (R^2^CV > 0.93) [102], minced lamb meat with pork [103], minced llama meat with horse meat (R^2^CV > 0.99) [104], vegetable protein, and soy flour in minced pork and beef (R^2^ > 0.78) [105].

Portable NIR spectrometers have been used for cost-saving but also for reliable monitoring of ham [98], beef [106,107], and routine analysis of Iberian pork [108]. Monounsaturated fatty acids, oleic acid, and saturated fatty acids could be predicted in wagyu beef carcass with R^2^ of 0.79, 0.71, 0.81, respectively, when a handheld fiber optic spectrometer was used [108].

### 3.5. Fish

Although there are not many studies about NIR spectroscopy applications in fish, the noninvasive advantages of NIR spectroscopy have been explored by some researchers for fish quality and the number of publications has increased in this field as well.

It is often difficult to differentiate between fresh and frozen/thawed fillets because fillets frozen below −60 °C do not show visual characteristics changes when thawed. In this regard, the classification of fresh and frozen fillet performed with a benchtop SMC spectrometer resulted in a high classification accuracy of 92% [109]. The outcome of this study is essential because fresh tuna is more expensive than thawed tuna, and it is important to prevent certain cases where frozen/thawed products are sold as fresh to deceive the consumer.

The pH, total volatile basic nitrogen (TVB-N), thiobarbituric acid reactive substances (TBARS), and ATP-related compounds (K value) of the bighead carp (*Aristichthys nobilis*) were predicted with high precision (R^2^ higher the 0.81) and low errors (RMSEP as low as 0.081) when a benchtop FT-NIR was used [110]. The study provides an alternative rapid approach for the determination of fish freshness.

For the analysis of sensory qualities, six textural properties (water holding capacity, hardness, resilience, springiness, chewiness, and shear force) of the silver carp fish (*Hypophthalmichthys molitrix*) were determined with NIR spectroscopy and predicted with high R^2^ (greater than 0.86 for all the parameters) and low error (RMSEP as low as 0.10) [110]. The determined sensory qualities are relevant in making consumer choices for fish consumption.

Another way of deceiving the consumer is done through the substitution of fish species. This was also investigated when a benchtop FT-NIR instrument was used for the authentication of Atlantic mullet and flounder fish fillets species with a 100% classification yield [111].

Handheld NIR devices have also successfully been used to distinguish fillets and patties of Atlantic cod with 100% correct classification in LDA [112].

### 3.6. Fats and Oils

Many varieties of vegetable oils, peanut oil, bean oil, rapeseed oil, sesame oil, oil-tea camellia seed oil, and olive oil, were investigated in the temperature range of 50–160 °C with two-dimensional correlation near-infrared spectroscopy (2D-NIR). In the 1666–1818 nm wavelength region, due to the elevating temperature, clear differences were observable in the 2D-NIR spectroscopy map. Thereby, the different oils could be distinguished directly by auto peaks and cross peaks [113].

Several other studies dealt with the detection of adulteration in diesel/biodiesel blends with vegetable oil. Back in 2015, one study using ultra-compact and FT-NIR spectrometers found that multiple linear regression (MLR) combined with successive projections algorithm (SPA) was the best modelling strategy. RMSEP values were 0.34% and 0.22%, and the limits of detection values (LOD) were 0.40% and 0.34%, for the ultra-compact and FT-NIR, respectively [114].

Casale and Simonetti (2014) comprehensively summarized the most important findings about NIR spectroscopy in the evaluation of olive oils. According to the review, NIR studies are primarily applied for adulteration detection, geographical origin prediction, quality parameter determination (including oxidative stability), and online process monitoring [115]. In a 2004 study, transmittance spectra of olive oil mixtures were analyzed in the 833–2500 nm range using a FT-NIR spectrometer to classify and quantify various adulterants. After multiplicative scatter correction (MSC), Savitzky–Golay smoothing, and mean normalization, the PLS models predicted corn, sunflower, soya, walnut, and hazelnut oil adulteration with R^2^ higher than 0.99 and error limits between ±0.57 and ±1.32% *w*/*w*. Additionally, the developed PCA models classified an unknown sample with an accuracy of nearly 100% [116].

Another study on olive oil counterfeiting, applying FT-NIR in the 1000–2500 nm range, revealed that the standard error of predictions ranged between 2.49 and 2.88% *V*/*V* for the olive-sunflower oil binary mixture, and 1.42–6.38% *V*/*V* for the ternary mixtures of olive–sunflower–corn oil. The R^2^ values of models estimating olive oil content were typically more than 0.99 [117].

To ensure the quality of fried foods, it is important to know how frying time and temperature affect physicochemical properties of the oils. Szabó et al. [118] measured conventional fat quality indices during prolonged heating of lard, and performed NIR-based calibrations using transflectance spectra recorded with a SMC spectrometer. Based on the cross-validation results, NIR spectroscopy was shown to be a rapid, solvent-free alternative for the estimation of acid value (AV) (R^2^ = 0.79) and *p*-anisidine value (pAV) (R^2^ = 0.77). In another study, Szabó et al. [119] introduced NIR spectroscopy models for predicting the deterioration level of four frying fats, such as rapeseed oil, sunflower oil, lard, and goose fat. Cross-validation precision for the amount of heat treatment (heating hours × temperature) exceeded 0.9, while calibrations for AV, PV, pAV, and total polar material (TPM) reached R^2^ over 0.8. Szabó et al. [120] combined NIR spectroscopy with a highly effective data reduction method, the polar qualification system, and developed models for identifying frying fat types, and for monitoring the quality changes of frying fats during long term usage. A Turkish study aimed to predict free fatty acid (FFA), TPM, viscosity, and smoke point of frying oils (refined hazelnut and peanut oils) by an FT-NIR spectrometer (780–2500 nm) and PLS regression after 5–35 h of dough frying. The results showed good correlation with the reference data. The r values of the standard quality parameters in the above listed order were 92.58, 94.61, 81.95, and 84.07, and the RMSEP values were 0.121, 3.96, 22.30, and 8.74, respectively (Öğütcü et al. 2012). Similar research was conducted on olive, sunflower, corn, and seeds oil after several hours of heating with and without foodstuff. In the measurements, a multipurpose analyzer FT-NIR spectrometer was utilized and the transmission spectra were recorded in the 666–2631 nm spectral range. Using a global model built on the spectra of all types of oils, the prediction of polymerized triglyceride content (PTG) was made with 2.28% *w*/*w* error [121].

Studies on canola oil have also shown that prolonged frying can be accurately identified with FT-NIR technique in the 1000–2500 nm wavelength range. The research used acid, peroxide, and carbonyl values as reference, then K-means, PAM, and hierarchical cluster analysis were performed to classify samples into harmful and less harmful categories [122].

Together, the above summarized studies corroborate the suitability of NIR spectroscopy in determining fat and oil authenticity by measuring quality parameters with high accuracy.

Furthermore, NIR spectroscopy proved to be an effective method for determining the level of degradation in frying oils.

### 3.7. Fruits and Vegetables

According to the summary of Cattaneo and Stellari (2019), among horticultural products, apples are most often tested with NIR spectroscopy [123]. Certain apple varieties are prone to internal diffuse browning. To detect this defect, beside some destructive methods, visible-shortwave NIR spectroscopy was used with different optical geometries. In the analysis, a handheld device using interactance mode (302–1150 nm), an “in-house” instrument employing partial transmission (302–1150 nm), and full transmission (600–973 nm) were used. The best results were obtained with the transmission arrangement, where R^2^ and RMSEP values of 0.83 and 0.63, were achieved, respectively, when predicting the defect score on a 5-point scale. Among the classification methods (PLS-DA, LDA, MD, SVM, SIMCA, MLoR), the best separation of acceptable fruits was determined with PLS-DA, classification accuracy higher than 95% and false discovery rate less than 2% were attained [124]. The FT-NIR spectroscopy in diffuse reflectance mode (1000–2500 nm range) proved to be successful when “Golden Delicious” apples from different altitudes and various apple cultivars were classified with PCA and quadratic discriminant analysis (QDA). Independent model validation resulted in a correct classifications rate of 87.5% and 86.3% for orchard elevation and for cultivar, respectively [125]. Other researchers also used the aforementioned methodology aiming to evaluate the long-term performance of calibration model for SSC prediction using the slope/bias (S/B) correction method on “Fuji” apples harvested between 2012 and 2018. The PLS model validations with five independent sets were realized in the 0.501–0.654% RMSEP range. The 15 selected wavelengths coupled with S/B correction could replace the whole analyzed spectra [126].

To investigate the feasibility of NIR spectroscopy in vegetable authentication, outdoor and greenhouse-grown bell peppers of different ripeness (green, red, yellow) were collected and analyzed in the 1600–2400 nm spectral range using a handheld microelectromechanical system-based device (reflectance mode) coupled with PLS-DA. The predictive models accurately discriminated the samples based on the cultivation conditions, where 89.73% and 88% correct classifications were obtained when unbalanced and balanced sets were used for model building. Classification accuracy between 88.28% and 91.37% was achieved when grouping was done according to ripeness. The dry matter and SSC predictions were realized with R^2^ values of 0.62, 0.63 and SECV between 0.66% and 0.75%, respectively [127]. A study by the same authors confirmed that NIR spectroscopy in static measuring position allows assessing some quality traits of freshly harvested Lamuyo peppers, such as color index (a*/b*), dry matter, SSC, and TA. The regression coefficients indicated 980 (water absorption), 1170–1360 nm (2nd overtone of C-H stretching) wavelengths important for parameter prediction [128].

Sweet corn is one of the most processed vegetables, the consumer acceptance of which is mostly determined by its sweet taste, which is given by the sugar content and composition. The soluble solid content of intact super sweet corn kernels was predicted using a SMC NIR reflectance analyzer in the 860–2500 nm range and synergy interval PLS (SI-PLS) after the best spectral pretreatment was selected. The best prediction was obtained when the 1349−1513, 1842−2005, 2005−2168, and 2337−2500 nm wavelength ranges were applied. To improve the predictive accuracy, the authors performed (CARS)-Si-PLS, hence the RMSEP amounted to 5.8292 mg/g and the correlation coefficient of the prediction set was equal to 0.8431 [129].

The NIR spectroscopy is widely and effectively used for testing horticultural products, both for quality property determination and authentication purposes. The estimation accuracy of the individual characteristics depends largely on what we would like to evaluate. The success of the investigation is dictated by the subject of the analysis, e.g., what physical properties a component has, in other words, what optical behavior or what sort of absorption it has in the NIR range.

## 4. Electronic Nose: Historical Background and Food Quality Assessment

**Figure 3:**
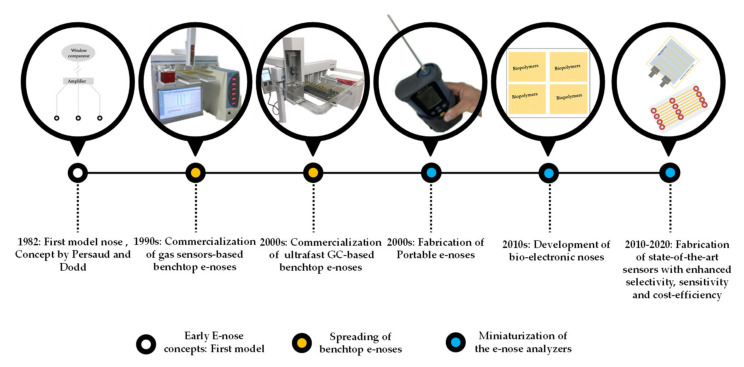
Overview of the evolution of the electronic nose. Self-developed figures based on authors’ own pictures.

Conceptualized by Persaud and Dodd [130] back in 1982, the E-nose has been defined as “an instrument, which comprises an array of electronic chemical sensors with partial specificity and an appropriate pattern-recognition system, capable of recognizing simple or complex odors” [131]. The multisensor technology started, however, 20 years earlier, in the 1960s, when the very first mechanical instruments were engineered to serve as simulators of the human olfactory abilities [132]. Ever since, the E-nose has undergone numerous conceptual changes. A historical overview of the evolution of the electronic nose with some of the main milestones has been presented in Figure 3, starting with the commercialization of the benchtop E-noses in the 1990s and followed by the fabrication of portable E-noses in the 2000s [133]. From the year 2010 till present (2020), novel sensors with advanced efficacies have been developed with innovative nanoparticle materials with increased and improved sensitivities and selectivity [134].

Principally, E-noses operate by converting existing volatiles of the studied matrices into electronic signals from the sensor array. The resultant digital output is then subjected to a data processing unit to extract the corresponding pattern.

Today, prompted by the need to analyze matrices of higher complexity, optimization efforts are still been targeted mainly at diversifying the types of sensors, the operating mechanisms, as well as the mastering of data mining and pattern recognition algorithms. This has led to a variety of instruments with numerous applications in the food industry [23].

Being a core constituent of the E-nose and a determinant of its efficiency, the sensors have dictated the type of instrument used. In this regard, those based on metal-oxide-semiconductor sensors (MOS), metal-oxide-semiconductor field-effect transistor sensors (MOSFET), mass-sensitive sensors, conducting organic polymers (CP), solid electrolyte sensors (SES), and fiber optic sensors have been the most predominant ones [29]. Acoustic wave chemical vapor sensors, existing ever since 1979 are also prominently used sensors. Based either on bulk acoustic wave or on surface acoustic waves, both of these techniques use the ultrasonic frequencies mainly from 1 to 500 MHz [135].

Whilst MOS operation is modulated by the change in conductivity caused by reactions of surrounding volatiles to adsorbed oxygen, the mechanism by which MOSFETs function is based on variations of the electrostatic potential [136]. Piezoelectric sensors, on the other hand, perform as a result of changes in the resonance frequency [137].

In designing such devices, basic criteria had to be fulfilled such as high sensitivity to chemicals of interest, cross-selectivity, stability, reliability, reproducibility, not to mention the robustness of the calibration models. Literature has shown, however, that each of these instruments has its corresponding assets and limitations. The most notable ones being the high temperature tolerance and low sensitivity to moisture of MOS sensors which renders them less prone to drift [137]. These oxide sensors, nevertheless, present a lower selectivity and a greater risk of poisoning by some weak acids. MOSFET sensors, although partly influenced by the temperature, have been touted to be highly robust [138]. The marked selectivity of piezoelectric sensors, on the other hand, is counterbalanced by their high sensitivity to fluctuating operational conditions (T° and humidity) whereas CP sensors, despite their good sensitivity and resistance to the poisoning effect, can have a considerably low reproducibility [139].

To overcome such shortcomings, some of the earliest suggestions were proposed by [140] who advised the applicability of the E-nose in controlled conditions for a thorough and accurate evaluation of the generated data. Others opted for using hybrid systems that incorporate the distinctive features of different sensor types [35]. Other researchers integrated the E-nose to gas chromatography for better performances [141]. Son et al. resorted to bio-electronic noses for a more rapid recognition of target components [142]. An equally promising option was explored by [143] and consisted of the fusion of the electronic nose and tongue, which, successfully resulted in better classification accuracies. Another particularly exciting prospect targeted the miniaturization of E-noses into portable devices for eventual application as routine on-site analysis tools [144]. Besides the olfactometry application of gas-chromatography, when human sensation and GC analysis is combined [145], headspace GC technologies are also emerging with ultrafast [146] or even miniaturized devices [147]. These fingerprinting technologies combined with a standardized database of signals of volatiles may help to overcome the burden of using a black-box type instrument where identification of fragrant substances is unachievable [148].

These findings attest to the adequacy of using such fingerprinting-based techniques in industrial settings where the lone dependence on panels and other conventional techniques exerts additional costs. Interestingly, studies have shown that outcomes of electronic noses could be correlated to descriptors of sensory panelists, E-noses could be more efficient than traditional methods and could further provide much finer grading of the studied food items [149,150].

Further enhancing the discriminatory features of the electronic olfaction systems by proper screening of array materials and designing drift-compensating apparatus could go a long way in increasing the robustness, selectivity, and sensitivity of E-noses thus their implementation for food quality applications [151].

### 4.1. Dairy Products

While the surge of the electronic nose (E-nose) inspired monitoring basic technological aspects such as the fermentation process of yoghurt, it certainly urged others to analyze other dairy products from different aspects [152,153,154,155]. Of the latter category, a mass spectroscopy related (MS-related) E-nose was used for the discrimination of *Latobacillus casei* species. The results revealed that the MS-related E-nose could differentiate the relatively similar strains, and predict the aroma of the products based on the results of the PCA projection [156]. An E-nose with commercial hybrid sensor array system containing 10 MOSFET and 12 MOS sensors was also applied for the discrimination between milk produced by healthy cows and cows infected with mastitis and proved its aptitude in such application. Other quality aspects such as the monitoring of cheese ripening [157] and the determination of the freshness of Crescenza cheese were assessed using an E-nose composed of MOSFET and MOS sensors with 100% accuracy of the classification of fresh, aged, and mature samples [158].

### 4.2. Sweeteners Including Honey

Origin determination of honey using an electronic nose has also spurred the interest of several researchers. An E-nose equipped with a MS detector using the SPME sampling technique was applied to classify honey according to their botanical origin: acacia, dandelion, chestnut, rape, linden, and fir. A 98% correct classification was obtained in PCA and DFA for the botanical origin [159].

Aimed at classifying honey of different botanical provenance using an E-nose consisting of six semiconductor sensors, a study revealed good classification accuracies that depended mostly on the instrumentation setup and applied temperature [160]. The best results were obtained for the following combination of parameters: 15 L/h volumetric flow, 35 °C of barbotage temperature, and acquisition time of 60 s.

In a Chinese study, samples from 14 different botanical and two different geographical origin were analyzed using an E-nose comprising metal oxide sensors (SnO_2_). The results showed that the E-nose was capable of discriminating the samples according to their geographical origin, and reasonable accuracies were also found for the botanical discrimination. Moreover, physico-chemical parameters such as fructose, glucose, Hydroxymethylfurfural (HMF), amylase activity, and acidity were predicted with coefficients of determination higher than 0.80 [161].

Similarly, the authors of [162] analyzed ziziphus honeys from Iran using an MOS sensors (MG and TGS) based E-nose and demonstrated its efficiency in successfully predicting the moisture (R^2^ > 0.9), pH (R^2^ > 0.9), ash content (R^2^ > 0.8), and free acidity (R^2^ > 0.9) of the honey samples.

### 4.3. Beverages

- Coffee

Brudzewski et al. [163] deployed a differential E-nose equipped with a matrix of MOS gas sensors to detect some common fraudulent practices in coffee. The study aimed at recognizing arabica coffee counterfeited with the cheaper robusta coffee. The built classification model provided with an average error of 0.21% recognition for the 11 classes of the mixtures of arabica and robust coffees. The authors concluded on the suitability of the method as a highly sensitive alternative to the usually adopted technique of liquid chromatography, that is both cumbersome and costly.

A variant in the work with electronic nose consisted of using 10 MOS sensors with different selectivity to volatiles to predict the roasting degree of coffee beans. Results revealed that analysis of the data of the E-nose with general least square regression combined with stepwise backward selection could be applied to determine the level of roasting (L*, mean density) and to detect the endpoint of the process. R^2^ values superior than 0.9 were obtained for the tested varieties (Brazilian, Indian, Vietnamese, and Costa Rican) [164].

In another study, the efficiency of an E-nose consisting of six metal oxide gas sensors in terms of differentiating different species of Robusta coffee was evaluated. Interestingly, while the electronic nose itself provided a classification rate higher than 90%, the fusion with E-tongue (seven chemical sensors with cross-selectivity) resulted in better accuracy [165].

Another application of the electronic nose using six MOS sensors was investigated for the discrimination of coffee samples dried at different conditions. Of all the studied samples, the hot-air dried and heat pump dried ones were the only ones to overlap on the PCA score plots [166].

Marek et al. (2020), on the other hand, analyzed coffee samples of different botanical origins using an electronic nose equipped with six MOS sensors and managed to separate Brazilian coffee samples from Guatemalan, Ethiopian, and Costa Rican using PCA plots varieties [167].

- Tea

Attempting to classify black tea samples of varying quality levels, the authors of [168] combined the data of the E-nose (equipped with five MOS sensors) to those obtained with an E-tongue. The classification accuracy of the 10-fold cross-validated PLS-DA models significantly improved from an initial value of 84.25% to 92.5%.

However, when coupled to two human panels, the rate of correct classification was slightly inferior and amounted to 90% when a predictive model derived of a five MOS sensor-based system (Figaro, Japan) was used for tea quality assessing [169].

In another study, PLRS analysis was used to predict the catechin, caffeine, polyphenol, and amino acid content of tea samples using E-nose (10 MOS sensors), E-tongue (potentiometric), and electronic eye. Based on the results of the E-nose, the best correlation was found in the case of caffeine quantification with an R^2^ of 0.609. The fusion of results of E-nose, E-tongue, and E-eye resulted in better separation and prediction accuracies (R^2^ 0.66–0.865) [170].

- Fruit juice

Application of the E-nose in juice analysis has mostly focused on its spoilage properties since these freshness criteria can function as predictors of their overall quality.

Reinhard et al. [171] applied a MOS base E-nose for the classification of 76 commercial citrus fruit juices and reported 96% correct classification of the tested juices.

An E-nose equipped with a MOS sensor array could detect bacterial concentrations as low as <10^2^ colony forming unit/mL of *Alicyclobacillus acidoterrestris* and *Alicyclobacillus acidocaldarius* in peach, orange, and apple fruit juices and was also able to classify bacterial contamination independently of *Alicyclobacillus* species with more than 78% of the variance in PC1 [172].

Detecting adulteration in juices is another important field of interest. Hong at. all studied seven data fusion approaches using PEN2 E-nose composed of 10 different MOS sensors and a potentiometric Alpha-Astree E-tongue. In the authentication of fresh cherry tomato juices adulterated with over-ripe tomato juice (0–30%), the data fusion of E-nose and E-tongue measurements presented promising results. CDA and library-SVM were found to ensure better classification whereas the best principle component regression (PCR) model fitting for pH and SSC estimation was realized with an R^2^ higher than 0.99 [173,174].

Ultra-fast GC based Heracles II E-nose was utilized to authenticate 100% orange juice mixed with 100% apple juice. Comparing different classification methods as HCA, classification tree, naive Bayes, ANN, and random forest classifiers. Random Forest classified the orange juice samples with almost 100% accuracy [175].

Multiple ester odorants (20) in apple juice were discriminated with significant differences using a MOS sensor array E-nose [176].

Sensory and postharvest qualities of sugarcane juice were classified with more than 97% variance in PC1 using an E-nose with 10 MOS sensors [177].

- Soft drinks

Microbial assessment of soft drinks is currently a pioneering area of interest in E-nose analysis. Contamination by *Alicyclobacillus* spp. in commercial flavored drinks was determined with 100% classification accuracy when an E-nose equipped with six thin film MOS sensors was used [178]. *Alicyclobacillus* spp. are thermoacidophilic, spore-forming bacteria that have potential health risks when consumed. Sodium carbonate powder was found to be very effective in absorbing moisture in the samples, which effectively improves the sensitivity and the stability of the E-nose sensors when Coca Cola, Pepsi Cola, Future Cola, and Sprite were accurately classified with 100% accuracy [179].

- Mineral water

Having anything but mild repercussions, the presence of water contaminants continues to be a thoroughly assessed criterion under the stringent restrictions of the World Health Organization (WHO). Geosmin (GSM) and 2-methylisoborneol (MIB), mainly produced by bacteria, are representative odor compounds and also indicators of contamination in water supply systems. Their presence was investigated with a newly developed E-nose composed of two nanovesicles and could detect GSM and MIB at concentrations as low as 10 ngL^−1^ [179]. An E-nose with 10 metal–oxide sensors, was also used to monitor the purification process in water treatment systems using water from the Nile river and showed significant differences in quality parameters: sulfates, nitrates, chlorides [2].

### 4.4. Meat

Gas sensors tend to have very broad selectivity, responding to many different substances so the E-nose is better adapted for meat and fish quality. It operates on the measurement of volatile compounds and, as such, requires very little or no sample preparation at all. An E-nose composed of six MOS sensors was used to determine beef freshness during 6 days storage at a temperature of 20 °C and relative humidity of 60% [180].

Similarly, an E-nose consisting of 10 different MOS sensors was used to predict the physical–chemical indexes: sensory scores, total volatile basic nitrogen, and microbial population of beef [181].

Other qualitative studies involved an E-nose with eight MOS sensors to determine beef decay with an accuracy 85.7%, 94.5%, and 96.2% for ANN, SVM, and KNN, respectively [182].

Haddi et al. used an E-nose consisting of eight heated MOS gas sensors to discriminate with 94.64% correct classification accuracy in SVM analysis samples consisting of goat, sheep, and beef stored at 4 °C for 15 days [183].

As per adulteration detection purposes, an E-nose with a sensor array of 10 different MOS sensors was used to detect and predict minced mutton samples tampered with 0%, 20%, 40%, 60%, 80%, and 100% pork [150]. This latter case is an important step in controlling food fraud.

### 4.5. Fish

Optimizing technological parameters such as time and pressure of applied vacuum is of paramount importance to ensure the desired quality of fish and fish products.

In this regard, in a pioneering study, an E-nose consisting of 14 gas sensors showed that the recommended time for storage (4 °C) of grass carp fillet to maintain its freshness under vacuum at 30 kPa was 10 days but at 50 kPa it was 12 days [184].

Another study deploying an E-nose with eight MOS sensors was targeted at determining fish decay and an accuracy of 85.7%, 94.5%, and 96.2% for ANN, SVM, and KNN, respectively, was achievable [182].

Horse mackerel, anchovy, and whiting fish species were discriminated with an E-nose consisting of eight metal gas sensors with 96.18% correct classification [185].

Species discrimination is an important nutritional and economical factor in the food industry but microbiological characterization is also of paramount importance. Changes in the signals collected from an E-nose with optical sensors showed that the dynamic supramolecular arrangement of liquid crystal molecules were in line with the total counts of mesophilic bacteria in tilapia fish species [186]. This is clear evidence of the potential application of an E-nose for monitoring fish deterioration.

More recently, portable E-nose systems have also been lauded for their ease of use as well as their ability to cut cost by performing onsite analysis instead of transporting samples to the laboratory. Examples of such benefits were reported by [187] for predicting fish freshness and by [112] for authentication of fish fillets.

### 4.6. Fats and Oils

The electronic nose also plays a prominent role in the authentication of vegetable oils. E-nose analysis (10 MOSFET sensors) combined with counter propagation artificial neural networks (CP-ANN) was conducted on extra virgin olive oils (EVOOs) originated from Garda (European Protected Denomination of Origin, PDO) and other regions to find a predictive classification model, in other words, to differentiate oils of different geographical origins. All the models were validated with commercial olive oil samples. According to the results, the classification model built on selected sensors appeared to be better than that built on the variables [188]. In another study, combining E-nose (10 MOSFET and 12 MOS sensors) and E-tongue (flow injection analysis with two amperometric detectors) means proved to be sufficient to determine the oxidation of EVOOs at different real-life storage conditions. During data analysis, PCA and LDA were used for predictive classification. In the course of validation, only one sample was misclassified [189].

Besides extra virgin olive oils, expensive edible oils are among the recurrent targets of food fraud. The authentication of sesame oil, in particular, is still an issue. For this consideration, a PEN2 E-nose (10 MOS gas sensors) was employed to detect maize oil adulteration (10–90%V/V). The authors found that the most efficient feature extraction method for linear discriminant analysis (LDA), probabilistic neural networks (PNN), and general regression neural network (GRNN) was Fisher linear transformation (FLT), while for back propagation neural networks (BPNN) was stepwise linear discriminant analysis (Step-LDA). The degree of misclassification was lowest at LDA. Outstanding results have been achieved when the adulterant concentration was predicted with BPNN (r = 0.998) and GRNN (r = 0.996) [190]. In another study by the same authors, in addition to sesame oil, the detectability of maize oil adulteration in camellia seed oil was investigated. Compared to principal component analysis (PCA), linear discriminant analysis (LDA) proved to be more effective to distinguish adulterations. Prediction accuracies of 83.6% for camellia seed and 94.5% for sesame oil were achieved when canonical discriminant analysis (CDA) was applied. Less precise results were typical for camellia seed oil (R^2^ = 0.842), and promising results were obtained for sesame oil (R^2^ = 0.998) when ANN was used to predict the adulterant concentration [191].

Lipid oxidation, a quality defect that takes place continuously, but to varying degrees from food processing to storage, seriously reduces the nutritional value of fats. In assessing this kind of phenomena, Triyana et al. (2015) reported the development of a portable E-nose using low-cost dynamic headspace and commercial metal oxide gas sensor array. In the mentioned research, vegetable oils (sunflower, grapeseed) and animal fats (chicken, mutton, porcine) were classified. The PCA evaluated the volatile fingerprint of all samples, based on which the clusters were separated. The first two PCs described 91.1% of the total variance [192]. When coupled to multivariate data analysis (cluster analysis, PCA, LDA), a PEN3 portable EN, consisting of 10 MOS-type sensors, was applied to evaluate lipid oxidation in a variety of edible oils (olive, peanut, soybean, rapeseed, camellia, corn, sunflower, linseed, and walnut). Slightly better results were obtained with LDA, whereas both the recognition rates for calibration and validation models were 100% [193].

### 4.7. Fruits and Vegetables

The E-nose has proven to be a very effective method in fruit identifications, ripeness assessments, and quality parameter estimations [194]. Many E-nose applications in the literature are destined, besides quality control, to monitor the ripening process and detecting mechanical damage of fruits and other vegetables during storage, from harvest to processing. These kind of studies are possible due to the formation, transformation, and decomposition of some of the volatile compounds contained by fruits or vegetables. [195]

Berna et al. [196] used quartz microbalance based E-nose and mass spectrometry (MS-nose) based E-nose to monitor the aroma profiles of two tomato cultivars (Tradiro, Clotilde) during shelf life. The MS-based E-nose showed more sensitivity, clear distinction between cultivars, and indicated an evident alteration in aroma profiles.

In a series of experiments, Chinese researchers conducted an E-nose based analysis for the freshness of fresh-cut green bell peppers during 9 days of storage using a commercial E-nose instrument (iNose) equipped with 14 MOS sensors. The hierarchical cluster analysis (HCA) showed the best separation between fresh and spoiled vegetables. Additionally, good correlation was achieved when aerobic plate count was quantified with PLS (R^2^ = 0.9783, RMSE = 0.3317) [197]. The same group of authors reported good results when monitoring fresh-cut broccoli during storage with both SPME-GC–MS and electronic nose (14 MOS gas sensors). The traditional analytical results (e.g., mass loss, chlorophyll content, malondialdehyde content and membrane permeability, aerobic plate count, sensory) were consistent with the electronic nose responses. PCA, HCA, and CDA could discriminate the fresh, medium fresh, and spoiled samples with high accuracy (100% correct classification) [198].

In one of the recent studies, a portable commercial PEN3 E-nose was used to detect and recognize moldy (*Penicillium expansum*, *Aspergillus niger*) “Golden Delicious” apples. The data corresponding to the most correlating sensors were processed by LDA, BPNN, SVM, and radial basis function neural network (RBFNN), where the best results were obtained with BPNN and the prediction accuracies varied between 72% and 96.3% [199]. Additionally, the portable commercial PEN3 was employed to monitor total soluble solid (TSS) and titratable acidity (TA) of litchi fruit exposed to varying degrees of mechanical damage (intact, mild, severe) and different storage conditions (room and cold temperature). The results were destined to reach better post-harvest management [200]. The detectability of fruit damage was also examined in yellow peaches using an in-house developed E-nose consisting of 14 Figaro TGS series sensors. The results, carried out using UVE-LS-SVM LS-SVM, showed that the correct answer rate was 93.33% at 24 h after the compression damage [201].

## 5. Electronic Tongue: Historical Background and Food Quality Assessment

The great advancement of the application of cross-selective gas sensors in the E-noses initiated the researchers and engineers to look for alternatives to analyze liquid samples similarly.

**Figure 4:**
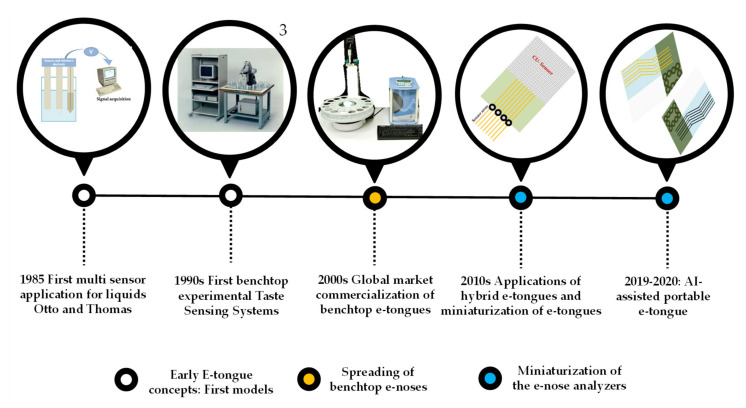
Overview of the evolution of the E-tongue. Reproduced with permission from Intelligent Sensor Technology, Inc. [205] (2020)^3^. The rest of the figures are self-developed based on authors own pictures.

A historical overview of the evolution of E-tongues with the main turning points of its evolvement is presented in Figure 4. Otto and Thomas proposed first in 1985 the application of liquid sensors in an array for analysis of different liquid samples. Then a few years later the pioneering concept of taste sensors was initiated by Hayashi et al. in 1990 [202] and have evolved, owing to technological advances, into highly sophisticated devices. As a result of the very active research in the development of liquid sensor array systems in many different countries in the 1990s the global market commercialization of E-tongue devices was possible in the millennium. By definition, an electronic tongue is an “analytical instrument including an array of non-selective chemical sensors with partial specificity to different solution components and an appropriate pattern recognition instrument, capable to recognize quantitative and qualitative compositions of simple and complex solutions” [203]. Fundamentally, E-tongues can transform the molecular information that is contained in the evaluated food items into visual patterns, representative of taste qualities but the main principle of operation often depends on the design of the E-tongue. In designing E-tongues, the differential array of sensors has been tailored depending on target samples by following varying operational modes. Of these instruments, the most prominent ones operate based on electrochemical, enzymatic, optical, and mass interactions [204].

Potentiometric E-tongues, namely those comprising ion-selective electrodes (ISEs) have been majorly used due to their cost efficiency, flexible set-up, and high selectivity. Their potential can, however, be highly affected by their temperature dependency and interferences caused by the adsorption of certain components [17].

Voltammetric sensors have been engineered for measurements of aqueous redox-active constituents and for such applications they were proven of great selective ability as they were capable of fingerprinting substances at low detection limits. Nonetheless, challenges pertaining to temperature-caused fluctuations and surface degradation restrict their applicability [206].

First introduced by Riul et al. [207], impedimetric E-tongues constituted another major milestone in artificial taste sensing that eliminated the need of referring to a standard reference electrode and comprised electrodes with specific chemosensitivity.

Operative in absorbance, fluorescence, and reflectance modes, optical sensors permitted the analysis of samples that were rather undetectable by electrochemical sensors. Such instruments, however, require specific preparation and can be subject to signal disturbances.

Mass sensors, on the other hand, function based on piezoelectric effect and present a promising alternative to other devices, owing to their sensitivity, robustness, and swift response read-out.

Assuredly, the surge of all these high-precision technological tools represents a breakthrough in analytical testing. With assets such as rapid determination of dissolved and volatile food constituents as well as accurate classification with direct measuring steps and minimal sample preparation [208], E-tongues, were shown to equal or outperform conventional methods. In the work by Escuder et al. [206], results obtained by artificial sensors were well-correlated to those given by panelists, more objective and less prone to toxicity. Additionally, compared to biological detection, the sensitivity of these artificial systems can be much better, more accurate, and can also be used effectively in fields where e.g., human qualification is not feasible [209].

Despite the wide range of available measurement approaches most of the published E-tongue applications deal with electrochemical sensors most commonly using either the potentiometric or the voltammetric detection methods. Resorting to multisensor analysis has not been devoid of limitations. Most of which are associated with memory effects, cross-contamination, drifts, and short-term validity of calibration models, among others. Of those who attempted to circumvent these impairments, the authors of [210] managed to successfully extend the calibration lifetime by applying univariate single sensor standardization corroborating the potential application of E-tongues in more complex applications. In [211], on the other hand, the authors developed a protocol for response standardization where the calibration transfer is applicable between arrays of differing sensors avoiding the need to perform laborious analytical analysis involving a large set of representative samples. Another way of enhancing the cross-selectivity of the sensors has been envisaged by Parra et al. [212], who diversified the coating materials of the electrodes including chemo-sensitive polypyrrole, metallophthalocyanine, and perylene derivatives. Since drift is one of the major hindrances precluding the wider application of E-tongues, various drift correction methods have been developed, that could substantially improve the long-term applicability of potentiometric E-tongue sensors [18]. Other approaches and their demonstrated performances have been extensively reported by [213]. Due to the improvements in electronics, measurement techniques, and signal processing methods along with the ever more sophisticated mathematical algorithms in the last decade, the miniaturization of the E-tongue has become possible. As pattern recognition devices that approximate human taste perception capacities, E-tongues have revolutionized traditional foodstuff assessment. A very promising variant in the work with E-tongues involves applications that go beyond the identification of basic tastes to resolving some food adulteration issues that have plagued both the research community and food practitioners. The following sections outline some of these applications.

### 5.1. Dairy Products

In the analysis of dairy products, the E-tongue has been a method of choice for many researchers. For instance, Dias et al. [214] tried to detect the adulteration of goat milk with bovine milk using a potentiometric E-tongue. Results of the model built for the discrimination of goat, cow, and goat/cow mixtures showed that the E-tongue could discriminate them with satisfactory accuracy with total classification recognition and prediction abilities of 97% and 87%, respectively.

In another study, the voltammetric E-tongue was applied to detect antibiotic residues in milk. Six antibiotics at four concentration levels were analyzed by four statistical techniques (PCA, DFA, PLS regression, and LS-SVM) and the results showed that all of the studied concentration levels could be discriminated from each other based on the results of the DFA model. The amount of the antibiotics was equally quantified with a quite high correlation (R^2^ > 0.9 for all of the antibiotics) [215].

As per [216], they have demonstrated that the voltammetric E-tongue, coupled with multivariate methods, was efficient for urea-tampered milk discrimination with accurate classification rates of 100% and 88.9% for calibration and prediction sets, respectively.

Wei et al. [217] investigated the capability of monitoring the quality attributes of yoghurt samples during fermentation, ripening, and storage by deploying the voltammetric E-tongue. The obtained results showed that the E-tongue was a promising method for tracking the state of the ripening of yoghurt and for predicting the acidity, the viscosity, and fermentation time of the samples. When subjected to PLS regression or SVM, R^2^ values higher than 0.9 were achieved.

The potentiometric E-tongue also proved its effectiveness when used to analyze the ripening of Cheddar cheese and to predict its sensorial characteristics. The findings showed that the ET could predict the sensory properties of the Cheddar cheese all along the 12-month storage period. Moreover, all the periods were classified correctly during training, however, the prediction accuracy decreased for the 5, 10, and 11-month-old samples to 67%, 67%, and 33%, respectively, after validation [218].

### 5.2. Sweeteners Including Honey

An equally important application of potentiometric E-tongues involved the determination of sugar concentration in different sugar solutions.

When testing solutions containing glucose, fructose, and sucrose in concentrations ranging from 0.3, 1, 2, 5, to 8 g/L, the concentration of the sugars were successfully predicted (R^2^ > 0.9) using the E-tongue data obtained with cross-selective lipidic polymeric membranes [219].

The detection of potential counterfeiting of honey is another focal challenge of the food industry. One of the commonly used methods of adulteration is done using sugar syrups [220] which could be directly mixed with the honey samples or indirectly fed to the bees during the collection period. In this regard, the authors of [221] used a voltammetric E-tongue to investigate the addition of glucose, inverted sugar, and inulin syrups to honey samples. The adulterated and authentic samples were rightfully classified with accuracies exceeding 90%. In [222] the authors equally reported a 100% recognition of honey adulterated with glucose and saccharose syrups using a voltammetric E-tongue. Identifying the origin of honey samples is a challenge that is often dictated by the geographical origin, climate, storage conditions, and processing [223]. This being said, using an Impedimetric electronic tongue enabled the successful discrimination of bupleurum honeys from lavender honeys [224]. Additionally, the botanical origin of acacia, linden, sunflower, honeydew, and multifloral honey were identifiable after processing the data obtained by a voltammetric electronic tongue using PCA and LDA analysis. What the results have shown is that the application of the four electrodes (gold, silver, platinum, and glass) provided the best results with 100% accuracy for training and 90% for validation. PLS regression was used to predict the pH, free acidity ash content, and color of the samples, which resulted in R^2^ > 0.9 after validation and R^2^ > 0.7 for ash and color parameters such as C*, a*, and b* [225].

A voltammetric E-tongue was also used to discriminate honeys from various botanical origins from Romania, showing 92.7% and 85.4% classification accuracy for training and cross-validation, respectively [226]. The potentiometric E-tongue seemed to be a promising method as complementary analysis of pollinic concentration of white, amber, and dark honeys [227].

The classification of chestnut, canola, acacia, and sunflower honeys from different botanical and geographical origin from Hungary was also assessed using a potentiometric E-tongue with high classification accuracies of 92.1% and 91.8% for training and cross-validation for the botanical origin, and higher than 90% for the geographical origin of the samples per honey type [228].

Likewise, the authors of [229] could discriminate multifloral honey according to their geographical origin from three countries using a voltammetric E-tongue based on their PCA results. Using the data of the ET, they managed to predict some physicochemical parameters of honey such as electrical conductivity, moisture content, color, fructose, and glucose content. While the sugar content of the samples could not be predicted, the remaining parameters could be determined with R^2^ higher than 0.8.

### 5.3. Beverages

- Coffee

An important aspect of coffee analysis revolves around ensuring the absence of fraudulent practices. In this regard, the Food Fraud Reports of the Joint Research Centre of the European Commission reported numerous cases of adulteration characterized either by mislabeling, origin masking, substituting high quality beans with low quality ones, as well as adding foreign materials such as the particles of the coffee as sticks and husk [230,231,232] and other noncoffee originated products such as brown sugar, chicory, and some grains [233]. Both the voltammetric and potentiometric E-tongues were proven efficient when used to classify pure authentic coffee samples from those adulterated with husks and sticks (R^2^ > 0.9 for the prediction of the adulterants) [233] and to classify the variety of Robusta coffee (95.2% classification accuracy) [165], respectively.

In another interesting study, different drying methods of coffee samples were investigated and analyzed using the E-tongue. The PCA of the E-nose, potentiometric E-tongue, and HS-SPME-GC- MS were able to effectively discriminate the coffee samples depending on the used drying processes [166].

Numerous studies have shown the importance of the geographical origin in conferring distinctive quality attributes to the coffee; notwithstanding, traditional techniques of determining this criterion are quite cumbersome. Alternatively, the authors of [234] attempted to determine the geographical groups of Arabica coffees from Colombia using a mini E-tongue comprising a polymeric sensor array with PPy modified using different counter ions. The acquired results showed the capability of the mini-E-tongue for the geographical based separation of the different coffee samples using PCA.

- Tea

When applying an E-tongue consisting of five noble metal electrodes to evaluate black tea samples of different quality grades, Banerjee et al. [168] demonstrated that a clear separation of the sample groups according to their quality was achievable. High classification accuracy amounting to 86.75% was obtained when PLS-DA analysis was used after 10-fold cross-validation.

In a similar pursuit, six different grades of tea were analyzed using a potentiometric E-tongue. The PCA results showed a pronounced separation tendency of the different grades. The catechin, amino acid, polyphenol, and caffeine content was predicted using PLSR, where the result of the testing set showed the best correlation with catechin content of 0.799 R^2^ [170]. According to [235], the cyclic voltammetry signals acquired with a portable E-tongue system and evaluated with chemometrics can be successfully used to predict the total theaflavins content in black tea samples. Hence, after the selection of the most effective variables, a determination coefficient R^2^ of 0.8302 was obtained.

- Fruit juices

Martina et al. [236] employed a voltammetric E-tongue for the analysis of fruit juices. The group of researchers, attempting to tackle drift issues of typically used electrochemical arrays, opted for poly(3,4-ethylenedioxythiophene) based electrodes. The purpose of the study was to investigate the suitability of such electrodes in complementing and/or substituting conventional ones, when applied to differentiate different juices.

In a combinatory manner, different assortments of electrodes were considered by interchangeably mixing bare and modified sensors. The authors concluded that the developed electrodes performed better than their bare counterparts in terms of cross-selectivity where the PLS-DA models classified with 100% accuracy the juices obtained from the same fruit, that were sourced from various brands. Moreover, cleaning these poly(3,4-ethylenedioxythiophene) based electrodes, between subsequent measurements was smoother.

In the qualitative and quantitative assessment of strawberry juice, Qiu et al. [143] resorted both to the separate use of a E-tongue or E-nose and their fusion. Recognizant of how impactful processing steps can be on the quality of the final product, the study was aimed at evaluating the effect of alternative processes (microwave pasteurization, freeze-thawing, temperature short time pasteurization, and steam blanching) on some juice quality parameters. The latter consisted of vitamin C content, total soluble solids pH, and total acid content.

What the results have shown is that the alpha-Astree potentiometric E-tongue had a better discriminating ability than the E-nose, both quantitatively and qualitatively. The fusion of the two multisensory systems further enhanced the accuracy of quality prediction with R^2^ values amounting to 0.9834 and 0.8959 for the calibration and validation, respectively.

- Soft drinks

Tonic water, a trending soft drink, owes its characteristic bitterness to quinine. On this account, examining their temporal changes during processing steps can be effective in predicting the bitterness, hence the quality of produced drinks. Since the commonly adopted flavor sensors, electrochemically operated, can easily fluctuate due to foulness and impurities, the authors of [237] utilized molecularly imprinted polymer coated electrodes for their study. The developed piezoelectric E-tongue had, not only good repeatability with a relative standard deviation inferior to 5% but also a good sensitivity of 2.04 mg/L when quantifying quinine. Most importantly, the results were comparable to those of panelists and only minor interferences emanated from sucrose.

With a growing inclination towards healthier drinks, the sugar content of marketed soft drinks can easily promote or deter their consumption. In this regard, Dias et al. [238] employed a lipidic/polymeric membrane-based E-tongue for an estimation of the glucose and fructose and respective R^2^ of 0.84 and 0.96 were achieved in different types and brands of soft drinks based on multiple linear regression and partial least squares regression models.

The system also allowed the accurate separation of the drinks depending on contents of orange, mango, peach, and pineapple fruit juices added in quantities lower than 4%, higher than 30%, and ranging from 14% to 30% and from 6% to 10%.

This type of instrument can, therefore, be a cost-effective promising tool offering a subjective evaluation of taste-conferring substances in this highly consumed type of drinks.

- Mineral water

Being the vital resource that it is, drinking water must comply with characteristic parameters defined by global legislative entities, and encompassing microbiological, biochemical, and even organoleptic quality standards.

Early applications of an E-tongue in the analysis of mineral water were reported by Legin and Rudnitskaya [239], who could differentiate between varying mineral waters with no considerable sensor drift. Within one group of samples, however, measured potential values were affected by the low minerals content. Despite that, the water groups were perfectly separated on the PCA plot. The same authors could fingerprint organic compounds that were randomly added to tested pure waters. This fingerprint translated into extreme principal component coordinates of the spiked water.

Additionally, by means of E-tongue analysis, determining quality criteria over the present decade has become accurately feasible. In this respect, Lvova et al. [240] employed a potentiometric E-tongue to trace 2-methyl-isoborneol (MIB) and geosmin (GE) pollutants in potable water. Their monitoring approach permitted the real-time detection of both low and high concentration ranges of 20–100 ng/L and 0.25–10 mg/L, respectively. In the absence of quantitative exigencies regarding the presence of these particular compounds in drinking water, mainly due to their unreported health-risks, this kind of study has been overlooked, hence the novelty of such undertaking, which is centered towards determining organoleptic quality of water.

### 5.4. Meat

The E-tongue has already been established to be better suited for liquid foods but current studies suggest its potential for meat and fish samples if the appropriate extraction methods are employed [27]. The E-tongue is advantageous in meat and fish analysis for multicomponent measurements because of its high selectivity, high signal-to-noise ratio, and various modes of measurements. Physicochemical and microbiological changes in fresh pork were studied with the potentiometric E-tongue composed of six sensors when pork loins were stored at 4 °C for 10 days [241]. pH, microbial analysis, hypoxanthine, inosine, IMP, and K-index were all predicted with high accuracies (R^2^) of 0.94, 0.88, 0.87, 0.87, 0.89, and 0.92 respectively. A voltammetric E-tongue using an array of seven working electrodes, a platinum counter electrode as an auxiliary, and an Ag/AgCl reference electrode was used to successfully discriminate (100% classification accuracy in SVM analysis) the origins of goat, sheep, and beef samples stored at 4 °C for 15 days [183]. In another study, the voltammetric E-tongue based on modified screen-printed electrodes with bisphthalocyanine and polypyrrole was used to detect ammonia and putrescine, used as markers of beef freshness in beef, with high accuracy (R^2^ higher than 0.95) and low error (RMSEP lower than 0.11) [242]. In their attempt to discriminate meat samples from different processing stages consisting of deep-frying, high-temperature boiling, and low-temperature braising [243] analyzed chicken breast samples for 5′-nucleotides and free amino acids deploying an E-tongue. Results showed that the inosine 5′-monophosphate (IMP), glutamc acid (Glu), lysine (Lys), and sodium chloride (NaCl) were the main compounds contributing to the taste attributes in the chicken breast samples.

### 5.5. Fish

Fish freshness is an important attribute in the food industry and has been studied with the voltammetric E-tongue for measurement of biogenic amines [244] and the potentiometric E-tongue for microbial populations [245]. The sensorial analysis was also attempted by Zhang et al. [246]. In their study, the peptides Tyr-Gly-Gly-Thr-Pro-Pro-Phe-Val were identified in the flesh of fish (*T. obscurus*) when a potentiometric E-tongue consisting of seven sensors was used to assess the contribution of the peptide to umami and sweet taste.

Likewise, the freshness of cod in 7 days storage treatment was successfully determined using a voltammetric E-tongue composed of eight metallic sensors [247]. Total viable count (TVC), mesophiles, enterobacteriaceae, hypoxanthine, inosine, IMP, and K-index were all predicted with high accuracies (R^2^) of 0.70, 0.78, 0.64 0.60, 0.60, 0.62, and 0.72, respectively.

The microbiological quality of Crucian carp fish was investigated by [248], who determined total viable count (TVC) with high accuracies and low errors using a potentiometric E-tongue with sensors.

Vacuum packaging, done through the removal of air in food packages, can be very proficient in extending the shelf-life of perishable foods such as fish fillets and fish products. In this context, a potentiometric E-tongue consisting of seven sensors was used to determine the freshness of grass carp fillets stored under vacuum (50 and 30 kPa) for 14 days at 4 °C [184]. As opposed to cumbersome and relatively expensive conventional analytical methods for identification of peptides or the determination of TVC, these studies show practical and efficient ways for rapid, accurate, and convenient analyses with the E-tongue.

### 5.6. Fats and Oils

Based on the current-potential relationship, voltammetry E-tongue tests were conducted mainly on high water containing samples (e.g., fruits, vines, etc.). Confronted with the challenge of testing low conductivity matrices the conventional way, Oliveri et al. (2009) worked out a methodology facilitating the direct voltammetric analysis of vegetable oils (maize, extra virgin olive oil) based on their quality and origin (Italian, Spanish). Their approach consisted of the addition of room temperature ionic liquids (RTILs) to oils to provide the intended conductivity. The voltammetric E-tongue measurements employed a two-electrode cell in a Faraday cage. In PCA, PC1 explained 58.14% of the variance that allowed a good separation between maize and olive oil. Classification with K-nearest neighbors (KNN) confirmed 100% discriminating ability. The method also completely distinguished the Italian and Spanish samples [249].

Potentiometric E-tongues have been frequently used in the authentication and qualification of extra virgin olive oils (EVOOs). Inspired by previous promising results, Portuguese and Spanish monovarietal EVOOs were discriminated according to the olive cultivar. The aqueous extraction with ethanol was indispensable, and solutions containing polar compounds were analyzed using two print-screen potentiometric devices with a total of 40 sensors. The most informative sensor signal profiles were selected employing a simulated annealing (SA) algorithm which proved to be a very robust method in terms of distinguishing the different cultivars when combined with LDA. This approach enabled all Spanish and three Portuguese samples to be discriminated with 61–98% sensitivity [250]. Another research team also used two print-screen potentiometric devices equipped with 20 sensors. On the research data, multiple linear regression (MLR) models were developed using SA to simultaneously quantify free acidity, peroxide values, ultra-violet light absorption extinction coefficients (K_232_, K_270_), and oxidative stability of Portuguese EVOOs exposed to 1-year storage and different lighting conditions. The authors concluded that quality parameters were predicted with adequate accuracy (R^2^ > 0.98 for leave-one-out and R^2^ > 0.96 for repeated K-fold cross-validation) [251]. A number of studies examining various Tunisian monovarietal olive oils of different quality grade also demonstrated the capabilities of potentiometric E-tongue coupled with SA-LDA in geographical origin assessment [252,253].

In another study, potentiometric E-tongue (12 solid state membrane sensors) coupled with PCA and PLS regression were used to evaluate some quality attributes (*p*-anisidine value (*p*-AV), peroxide value (PV), total tocopherol content (TT)) of rapeseed, sunflower, soy, peanut, and olive oils. The sample preparation had to be optimized before starting the measurements. The results demonstrated good promise for recognition and prediction of different oils. The R^2^ values of validation were between 0.67 and 0.89, and the RMSECV values of PV, *p*-AV, and TT were 0.5 meq/kg, 0.8 arbitrary units, and 10 mg/100 g, respectively [254]. Since the E-tongue is more suitable for measuring solutions and polar compounds dissolved in an aqueous system, it is difficult to test viscous, minimally conductive materials. In addition to the usual sample preparation, the addition of substances intended to dissolve the oils and to act as a supporting electrolyte is imperative. This is especially true for the above-mentioned voltammetric measurements.

### 5.7. Fruits and Vegetables

The consumer perception of horticultural products is influenced by the shape, texture, and taste (sweetness, acidity).

In the previous study of Beullens et al. (2008), the feasibility of a self-constructed (18 potentiometric sensors) and an Astree (7 ISFET sensors) E-tongue were compared when individual sugars, organic acids, and mineral contents were quantified in tomatoes. Six Belgian tomato cultivars were classified very well based on their taste profile. The PLS prediction based on the laboratory-made system demonstrated correlation coefficients higher than 0.97 and 0.87 for calibration and validation, respectively. However, the method could not predict some taste attributes (general sweetness, umami taste). The other E-tongue correlated well with glutamic acid, sodium and potassium, when correlations were higher than 0.95, and RMSE values were 0.1 in the course of validation, while for the sensory parameters, these values were between 0.7 and 0.94 and less than 1.22, respectively [255].

Research on different soybean cultivars for human consumption revealed that the eight sensor-based E-tongue distinguished the varieties similar to the trained panel. The sensory traits were in good correlation with the chemical components of the beans. The device was apt to classify the different cultivars based on their flavor profile [256].

In a 2013 study, the sugar content and firmness of nonclimacteric pears were evaluated with a self-developed voltammetric E-tongue composed of six working electrodes. The characteristic data were compressed into PCs and then PCR, PLS regression, and LS-SVM were performed to estimate the above-mentioned two quality attributes of five different cultivars of different geographical origins. The best results were obtained with LS-SVM when the coefficients of determinations were higher than 0.99 [257].

In the comprehensive sensory and physicochemical analysis of peanuts, high-oleic and conventional cultivars were evaluated and compared. The fatty acid composition and tocopherol contents were determined preliminarily. A major finding was that the taste scores of the high-oleic cultivar measured with an E-tongue were quite distributed to all tastes compared to conventional ones [258].

In their paper, Wu et al. [259] employed an electronic nose (10 MOS chemical sensors) and E-tongue (seven ISFET electrodes) to trace apple varieties according to geographical origin. PCA was used as a primary mapping. The LDA, SVM, and PLS-DA models were later developed for the discrimination based on the individual and fusion datasets. The authors concluded that more robust classification models could be constructed with data fusion, and thus the accuracy of discrimination could also be improved.

In many cases, the grafting of fruit and vegetable crops are carried out to increase resistance and/or yield of the plant. The impact of this procedure on quality properties requires deeper research. To expand this knowledge, grafted and nongrafted watermelons grown in different regions of Hungary (Békés, Jász-Nagykun-Szolnok, Fejér county) were analyzed with potentiometric E-tongue (seven ISFET sensors) to predict characteristic sensory attributes, concerning color, texture, juiciness, global scent, and taste. The results revealed that the cultivation circumstances determine mostly the quality factors, not the type of grafted rootstock [260]. Orange and green-fleshed melons were also studied using the same instrument when the effect of grafting and storage conditions on chemical composition and sensory profile were evaluated. The same potentiometric E-tongue measurements combined with LDA showed 100% separation between the flesh of different colors. Good correlations were found between some standard analytical parameters and E-tongue sensor signals with PLS regression models [80].

E-tongue studies on fruits and vegetables have indicated that they can be categorized with high accuracy according to different aspects based on their taste profile. The estimation of some quality traits is realized with varying accuracies, which depends to a great extent on the interaction of the matrix and the sensors. The E-tongues generally, react with greater sensitivity to samples containing more polar compounds.

## 6. Conclusions

Near infrared (NIR) spectroscopy, electronic nose (E-nose), and electronic tongue (E-tongue) have been widely applied in the food industry for quintessential quality control purposes. There has equally been an unprecedented improvement of the instruments using state-of-the-art materials for enhanced performance to keep pace with the ever-growing complexities of the food chain. Based on the commonly consumed food matrices defined by the Codex General Standard for Food Additives, this special issue paper shows that, in combination with chemometrics and multivariate data analysis, the instruments have exhibited a trifecta in the determination of multiple quality parameters in dairy products, sweeteners, beverages, fruits and vegetables, and meat and fish industries.

NIR spectroscopy has been in existence since the 1950s and is the oldest among the three instruments. Several studies focusing on improving the reliability of the technique have led to the development of different NIR instruments such as the interference filter spectrometers, scanning grating spectrometers, LED-based spectrometers, AOTF devices, diode array grating polychromators, and even handheld and miniaturized spectrometers. The E-nose is the second oldest among the instruments and has been in existence since 1982. Research developments have led to even more advanced E-nose versions such as the bio-electronic E-nose and portable E-noses with enhanced sensors for increased selectivity, sensitivity, and cost-efficiency. Predominant among these sensors are the metal-oxide-semiconductor sensors (MOS), metal-oxide-semiconductor field-effect transistor sensors (MOSFET), mass-sensitive sensors, conducting organic polymers (CP), solid electrolyte sensors (SES), and fiber optic sensors. The E-tongue has been in existence since 1985 and has been subject to massive improvements such as the development of advanced sensors, hybrid and miniaturized E-tongues, and AI-assisted portable E-tongues that can yield optimized analytical results. Many types of E-tongues have been developed based on the principle of operation, but the potentiometric and voltammetric E-tongues are the most dominant.

The instruments (NIR spectrometers, E-nose, E-tongue) could produce high accuracies for monitoring physical, physicochemical, and sensory parameters of food such as yoghurt, fruit and vegetables, vegetable oils, coffee, honey, milk, sweeteners, cheeses, meat (pork, beef, oxen, mutton, chicken, turkey), and fish (fish fillets and multiple species). They have been effective in the geographical classification of different animal species, water sources, and food products. For authentication purposes, they have been used to detect contaminants and predict adulteration of milk with water, peach juice with sugars, cherry tomato juices with overripe tomato juice and flavored drinks with sodium biocarbonate. In the meat industry, high correlations were obtained for the detection of minced beef with turkey meat, minced lamb meat with pork, minced llama meat with horse meat, and soy flour in minced pork and beef.

NIR spectroscopy is noninvasive and can be used for quality control purposes in all foods. Similarly, the E-nose can be applicable for both solid and liquid foods so long as enough time is given for the food volatiles to reach the instrument headspace. The E-tongue, on the other hand, is better suited for liquid analysis but this special issue paper showed that it can also be suitable for nonliquid foods if the appropriate extraction methods are used. The arsenal of chemometric and multivariate data analysis tools increases the reliability of the instruments for diverse analytical measurements. The emergence of handheld and miniaturized versions makes them very cost-effective and assures their unlimited potential for product development and food control in the industry.

## Figures and Tables

**Table 1:** Comparison of correlative analytical methods (near infrared (NIR) spectroscopy, electronic nose (E-nose), electronic tongue (E-tongue)) to sensory analysis and major conventional analytical methods (MS: mass spectrometry; GC: gas chromatography; PCR: polymerase chain reaction; ELISA: enzyme-linked-immuno-sorbent-assay). Self-developed concepts were adapted from Valle et al. [11], Bansal et al. [12], Huang et al. [13], Mæhre et al. [14].

	Conventional Methods							Correlative Analytical Methods		
Criterion	Sensory Analysis	MS	Chromatography	PCR	ELISA	Dumas	Soxhlet	E-Tongue	E-Nose	NIR Spectroscopy
Affordability	No	No	No	Yes	Yes	No	No	Yes	Yes	Yes
Technicality	Yes	Yes	Yes	Yes	Yes	Yes	Yes	No	No	No
Low detection limit	No	Yes	Yes	Yes	Yes	Yes	Yes	No	Yes	Yes
Portability	N.A.	Yes	Yes *	Yes	Yes	No	No	Yes	Yes	Yes
Reagents	No	Yes	Yes	Yes	Yes	Yes	Yes	No	No	No
Sample preparation	No	Yes	Yes	Yes	Yes	No	Yes	Little to none	Little to none	No
Selectivity	Yes	Yes	Yes	Yes	Yes	Yes	Yes	No	No	No
Specificity	Yes	Yes	Yes	Yes	Yes	Yes	Yes	Yes	Yes	No
Analysis method	Direct	Direct	** Direct	Direct	Direct	Direct	Indirect	Indirect	Indirect	Indirect
Maintenance	N.A.	Expensive	Expensive	Expensive	Expensive	Expensive	Expensive	Cheap	Cheap	Cheap
Rapid measurement time	No	Yes	No	Yes	Yes	No	No	Yes	Yes	Yes
Qualitative and Quantitative analysis	Yes	Yes	Yes	Yes	Yes	Yes	Yes	Yes	Yes	Yes

*: only for liquid and gas chromatography; **: when standards are used; N.A.: not applicable.
